# 2237. Enterbacterales Resistance Patterns for Extended-spectrum beta-lactamases (ESBL) Positive and Multidrug Resistance (MDR) Urine Isolates Collected and Tested from 295 Outpatient US Facilities in 2019

**DOI:** 10.1093/ofid/ofac492.1855

**Published:** 2022-12-15

**Authors:** Mauricio Rodriguez, Kalvin Yu, Vikas Gupta

**Affiliations:** Spero Therapeutics (Former Employee), San Antonio, TX; Becton, Dickinson and Company (BD), Franklin Lakes, New Jersey; Becton, Dickinson and Company (BD), Franklin Lakes, New Jersey

## Abstract

**Background:**

Approximately 3 million cases of complicated Urinary Tract Infections (cUTI) occur annually in the U.S. with over 80% being diagnosed in the community setting. Antimicrobial resistance (AMR) and MDR are a growing threat. ESBL producing Enterbacterales (ENT) alone have increased by 53% from 2012 to 2017. Agents commonly used for treatment are becoming ineffective due to higher baseline resistance, resulting in treatment failures and unnecessary hospitalizations.

**Methods:**

In total, 669,664 ENT (*E. coli*, *K. pneumoniae*, *K. oxytoca*, *P. mirabilis*) urine isolates were collected and tested from 295 US facilities (BD database) in 2019. All 30-day non-duplicate isolates collected were from adult outpatients (OPs). Antimicrobial susceptibility was done by each facility reference lab. The total number of isolates tested, and total non-susceptible (NS) isolates were tested and identified at the county level. Only counties with ≥ 30 tested isolates were reported. Those with < 30 isolates tested were indicated as < 1% NS (e.g., insufficient isolates tested). Counties without susceptibility results were populated to the nearest county either within or across state lines. We sought to examine both ESBL and ≥ 3 drug NS rates in the OP setting.

**Results:**

Among all isolates, ESBL + and ≥ 3 drug NS rates represented 8.5% and 4.9%, respectively. A total of 14 states (28%) had resistance rates ≥20% for ESBL producing ENT. MO had the highest rate (28.6%), followed by 24.7% for KY, NC, TN, VA, and WV. GA was at 23.7%, while AZ, NM and TX were∼ 21.2%, and FL at 20.2%. The remain states DE, MD, and NJ were all at 20% (**Figure 1**). MDR (≥ 3 drug NS) rates ≥ 10% were noted for 11 states (22%). States in rank order included: MO (20%), KY, NC, TN, VA, and WV all at 14.7%. KY ranged from 14.7% to 10.1%, whereas GA was at 11.5% with 10.7% for both AL and FL. The remaining states included CA and HI both at ∼10% (**Figure 2**).

**Figure 1.**
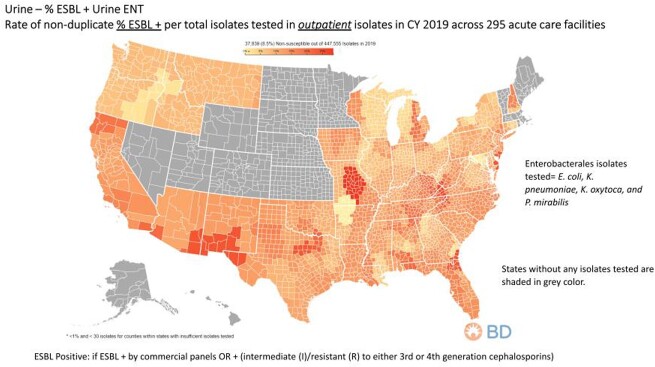

**Figure 2.**
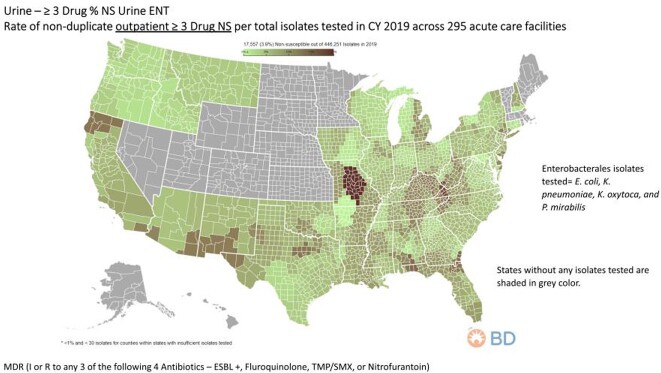

**Conclusion:**

Important regional differences are noted for ESBL + and ≥ 3 drug resistance to urine isolates in the OP setting complicating oral antibiotic therapy. Certain states with higher resistance rates require education for the guideline selection of appropriate antibiotics. The approval of newer agents that overcome AMR are warranted and offer an alternative to limited existing oral therapy.

**Disclosures:**

**Mauricio Rodriguez, PharmD, MS-HEOR, BCPS, BCCCP, BCIDP**, Spero Therapeutics: Employee **Kalvin Yu, MD, FIDSA**, Becton, Dickinson and Company: Employee of, and shareholder in, Becton, Dickinson and Company, and the company received funding from GlaxoSmithKline plc. to conduct this study|GlaxoSmithKline plc.: GlaxoSmithKline plc.-sponsored study 212502 **Vikas Gupta, PharmD**, Becton, Dickinson and Company: Employee of, and shareholder in, Becton, Dickinson and Company, and the company received funding from GlaxoSmithKline plc. to conduct this study|GlaxoSmithKline plc.: GlaxoSmithKline plc.-sponsored study 212502.

